# Mobile tablet-based therapies following stroke: a systematic scoping review protocol of attempted interventions and the challenges encountered

**DOI:** 10.1186/s13643-017-0620-6

**Published:** 2017-11-02

**Authors:** Michael Pugliese, Dylan Johnson, Dar Dowlatshahi, Tim Ramsay

**Affiliations:** 1 School of Epidemiology, Public Health, and Preventive Medicine, University of Ottawa, Alta Vista Campus, Room 101, 600 Peter Morand Crescent, Ottawa, ON K1G 5Z3 Canada; 2 Department of Medicine (Neurology), University of Ottawa Brain and Mind Research Institute, and Ottawa Hospital Research Institute, C2182 Ottawa Hospital Civic Campus, 1053 Carling Avenue, Ottawa, ON K1Y 4E9 Canada; 3 Ottawa Hospital Research Institute and Scientific Director at the Ottawa Methods Centre, University of Ottawa, Alta Vista Campus, 501 Smyth Rd, Ottawa, ON K1H 8L6 Canada

**Keywords:** Stroke rehabilitation, mHealth, iPad, Tablet computer, CVA, Disability

## Abstract

**Background:**

Stroke is a growing global epidemic limiting the ability of millions to function independently due to post-stroke deficits and complications. Although specialized stroke rehabilitation improves the recovery of functional abilities, accessing rehabilitation services has become increasingly challenging as the number of stroke survivors continues to increase and rehabilitation resources remain scarce. Mobile tablet-based therapies (MTBTs) may be a resource-efficient platform for providing stroke rehabilitation services. The feasibility and challenges of offering MTBTs to stroke survivors should be well understood before expensive, large-scale clinical trials are undertaken to study treatment efficacy.

**Method:**

A systematic scoping review will be conducted to describe attempted MTBTs following stroke and the challenges encountered by survivors and study staff. Studies of interest will evaluate MTBTs offered to adult stroke patients in response to post-stroke complications or deficits. Journal databases, gray literature sources, clinical trial registries, relevant organizational websites, and reference lists of eligible studies will be searched to identify suitable studies. Study characteristics, barriers to care, methodological challenges, patient-reported outcomes, and health outcomes will be extracted to describe MTBTs and understand the challenges encountered in context. Results will be presented using descriptive statistics, tables, figures, and narrative description to summarize the scope of the field.

**Discussion:**

Trends in MTBT feasibility and common challenges will be discussed to summarize major findings and highlight research gaps. Solutions to common challenges experienced by intervention participants and study staff will be proposed. Implications for the conduct of randomized clinical trials of MTBT efficacy and the appropriateness of a systematic review and meta-analysis of completed trials will be discussed.

**Systematic review registration:**

uO Research (http://hdl.handle.net/10393/35696).

**Electronic supplementary material:**

The online version of this article (10.1186/s13643-017-0620-6) contains supplementary material, which is available to authorized users.

## Background

### Rationale

#### The growing burden of stroke

Stroke is a global epidemic affecting millions worldwide. It is the second leading cause of death [1] and the third leading cause of disability globally [2]. The financial impact is also immense, incurring billions of dollars in healthcare costs annually [3]. This enormous burden has continued to increase with a reported 68% rise in the absolute number of first-time incidences of strokes and an 84% increase in stroke survivors worldwide in 2010 when compared to 1990 [4]. Disability and massive healthcare costs are caused by the wide range of post-stroke impairments and complications experienced by stroke survivors. Specialized stroke rehabilitation effectively improves recovery following stroke [5] with the greatest improvements occurring when therapy begins early post-stroke and is performed intensely [6]. Best practice recommends timely transfer of stroke survivors to well-staffed, specialized inpatient rehabilitation units with sufficient resources to offer the variety and intensity of therapy needed to reduce chances of death and improve functionality.

#### Accessing stroke rehabilitation services

Accessing early and intensive stroke rehabilitation is challenging. Rehabilitation is not consistently initiated in the acute setting [7], and only 16% of stroke survivors in Canada are transferred to inpatient rehabilitation centers when estimates suggest 40% of survivors would benefit [8]. In the USA, only 24% of patients are transferred to inpatient services and only after waiting an average of 27 days post-stroke [9]. Accessing outpatient and community care is also difficult [10] with not all patients having immediate access to outpatient and community rehabilitation services [11]. The poor availability of stroke rehabilitation services is thought to be due to a lack of therapists with expertise in stroke [7, 9–11]. Additionally, therapists reported being assigned between 10.5 and 56 beds per therapist in Ontario rehabilitation centers [12] suggesting current therapists are already overburdened and unable to provide the intensive therapy needed for improving recovery.

#### Improving the accessibility of stroke rehabilitation using mobile tablets

Over the past decade, there has been growing interest in harnessing technology to support stroke rehabilitation and provide adjunctive therapy. There is growing research suggesting the positive effects of gaming on neurological outcomes following stroke with home video game consoles and virtual reality in particular have been shown to improve upper limb function and performance on activities of daily living [13]. Mobile video games have become available for mobile tablets in the form of software applications (apps), and there has been growing interest in mobile tablet-based therapies (MTBTs) following stroke. MTBTs use apps running on mobile tablet computers to provide interventions to patients. MTBTs are separate from and do not include therapies delivered via smartphone, mobile phone, or non-mobile touchscreen tablet technologies which are considered different therapeutic platforms. There are a variety of apps either explicitly designed to offer therapy (Constant Therapy© for aphasia and cognitive impairments) or involving activities analogous to scenarios often used in stroke rehabilitation (memory and attention games, etc.). Survivors could use MTBTs to either supplement therapist-led rehabilitation or begin engaging early rehabilitation until therapist-led services are successfully accessed.

#### Why is it important to do a review now?

Despite their wide availability, relatively low cost, and technological power, much remains unknown about MTBTs including treatment efficacy. However, before attempting small- or large-scale randomized controlled trials (RCTs) of treatment efficacy, the feasibility and challenges of offering MTBTs following stroke should be well understood in order to improve the chances of conducting successful studies. The study of MTBTs following stroke is still relatively new, and to the best of our knowledge, there is no systematic review summarizing attempted MTBTs. This systematic scoping review will be the first in the topic area and can provide answers to key questions regarding the feasibility and challenges of MTBTs following stroke while also describing the breadth of the field, identifying gaps in research, and informing the conduct of future RCTs and the appropriateness of meta-analysis of completed RCTs.

### Objective

The objective of the study protocol is to review the evidence for mobile tablet-based therapies (MTBTs) following stroke.

#### Research questions

1. What are the characteristics of attempted MTBTs following stroke in terms of targeted deficits and method of administration?
2. What barriers or adverse events related to the administration of MTBTs following stroke have been encountered by researchers, clinicians, caregivers, or participants?
3. What methodological challenges have been faced by studies of MTBTs following stroke?

## Methodology

This protocol was developed primarily with the assistance of a published guideline for scoping reviews [14] and with PRISMA-P (Additional file 1) when necessary and appropriate [15].

### Inclusion/exclusion criteria

Inclusion criteria (must meet all):

1. Adult stroke survivors (18 years or older) of any type (ischemic/hemorrhagic) or stage (acute/chronic) in any setting.
2. Stroke survivors interacting with a mobile tablet (not a smartphone, mobile phone, or non-mobile touchscreen tablet) in response to a post-stroke deficit or complication for therapeutic purposes.

Exclusion criteria (exclude if meet one or more):

1. The mobile tablet is primarily used by someone other than the stroke survivor for purposes unrelated to tablet-based therapy support.
2. The mobile tablet is used primarily for purposes other than therapy.

### Criteria explanation and elaboration

#### Population

Only studies involving adult stroke survivors will be included; children are a separate population outside of the scope of the proposed review. There are no restrictions with regard to stroke type or stage as the field is expected to be heterogeneous in this regard. Studies involving a mixture of stroke and non-stroke participants will be included only if they have separately reported data about stroke participants.

#### Intervention

We define MTBTs as patient-driven therapies in which participants interact via touch, speech, or movement with mobile tablet devices in response to a deficit or complication. The tablet device should be the primary method of therapy delivery; however, therapies involving peripheral devices (devices other than the core tablet unit itself including smartphone, robotics, sensors, etc.) will be included if the mobile tablet is clearly the primary platform for delivering therapy. MTBTs do not include smartphones, mobile phones, or non-mobile touchscreen tablets. This distinction has been because mobile tablets offer the unique benefit of having a large touchscreen interface that is likely easier for stroke survivors, who often suffer from motor and cognitive deficits, to manipulate while still remaining easily portable. This portability could allow survivors to bring their MTBT with them across their continuum of care from the acute hospital setting shortly after their stroke to their discharge destination. The use of tablets as assistive devices by clinicians for the administration of therapy, or by patients for the primary purpose of screening, assessment, or data collection does not constitute a MTBT. Tele-rehab programs using tablets solely as a method of videoconferencing with participants will not be included as the therapy is not truly mobile tablet-based; the tablet is simply acting as a means of providing traditional therapist-driven treatment.

#### Context

There are no restrictions related to context as we are interested in interventions performed in all settings and geographical locations, administered in all languages, and delivered by all types of therapists or non-therapists. However, only English-language publications will be considered due to expensive translation costs.

#### Comparator(s)

There are no restrictions related to comparators (standard treatment, workbooks, desktop or laptop computers, smartphones, etc.) as we are interested in describing all attempted MTBTs following stroke regardless of comparisons to other therapies.

#### Outcomes

There are no restrictions with regard to study outcomes as we are primarily interested in attempted interventions, therapy barriers, and research challenges. However, considering the study goals and research questions, we are interested in study outcomes including but not limited to barriers to care, adverse events, protocol deviations, Research Ethics Board issues, recruitment rate, adherence rate, retention rate, and patient evaluations of MTBTs.

#### Study designs

There are no restrictions with regard to study design: case studies/series, prospective and retrospective cohort studies, and randomized or non-randomized controlled trials of all designs will be included. There will be no restrictions with regard to study timing as it is expected that studies will substantially vary in length and timing. Study protocols and conference abstracts will only be included if they contain pilot or preliminary results from a study whose data are otherwise unavailable from a full-study manuscript. Included studies will be clearly marked as full-text articles, protocols, and abstracts, accordingly.

#### Other restrictions

As mentioned above, the included studies will be restricted to those written in English. Although the first modern tablet computers were introduced in the early 2000s, the surge in popularity of tablet computers with the release of the first Apple iPad© in 2010 is well known. Additionally, the mobile tablet computers which will eventually be included in future randomized controlled trials will likely continue to improve upon the capabilities of their counterparts presented in this review. Therefore, in order to collect information that will be most relevant to information future RCTs, searches will be restricted to include studies between 2010 to present.

### Information sources

#### Preliminary search

A preliminary search of the literature using key terms related to stroke and mobile devices in MEDLINE (OVID interface) yielded a number of studies meeting our inclusion criteria. These articles were used to identify key words and build a search strategy with the aid of a health information librarian. One study author (MP) piloted the search strategy in MEDLINE to ensure the strategy successfully re-identified the studies used to build the search. The search strategy successfully identified these papers, and no further modifications were made to the strategy except those necessary to adapt the strategy to different database search interfaces.

#### Database searches

The following six databases will be searched: MEDLINE (OVID interface), EMBASE (OVID interface), PsycINFO (OVID interface), CINAHL, Cochrane Database, and Web of Science.

#### Additional information sources

1. A snowball search of relevant articles and reviews identified by the database search.
2. Organizational websites: Aphasia.org, American Stroke Association webpage, Heart and Stroke Foundation webpage, and Stroke Engine.
3. Clinical trial databases will also be searched for completed and ongoing studies: ClinialTrials.gov, the WHO International Clinical Trials Registry Platform, EU clinical trials database, and ISRCTN.

#### Gray literature search

A gray literature search will also be performed in order to find unpublished material using Google Scholar, the ProQuest Dissertation and Theses Database (Global and UK & Ireland), and the OpenGrey European gray literature database. After a preliminary search of Google Scholar and ProQuest Dissertation Global, it was decided searches would be limited to the first 200 results as a compromise between conducting a robust search and exhausting resources as search results beyond the first 200 results appeared to be irrelevant [16].

### Database search strategy

The search strategy presented in Table 1 will be used to search databases with Ovid interfaces and adapted to search databases using other search interfaces. All index database searches were restricted to between the years 2010 and present and English language.

**Table 1:** PRISMA-P checklist

Search terms	
1.	exp Stroke/
2.	exp cerebrovascular disorders/
3.	(stroke* or cerebrovascular* or cerebral vascular or CVA*).tw.
4.	((cerebr* or brain) adj3 infarct*).tw.
5.	1 or 2 or 3 or 4
6.	(mobile device* or mobile computer* or handheld computer* or tablet*).tw.
7.	(ipad* or galaxy tab* or surface pro*).tw.
8.	6 or 7
9.	5 and 8

### Study records

#### Data management

Database search results will be downloaded and imported to reference management software (Endnote™ X8) in order to search for duplicates [17]. After duplicates have been removed using software and manual identification, the database results will be uploaded into Covidence©, an online systematic review manager, where all title/abstract and full-text screening will take place [18].

#### Selection process

Two authors (MP and DJ) will independently screen collected articles in a two-stage process with the assistance of an article screening form (Table 2): co-screeners will (1) screen study titles and abstracts returned by database searches for potentially eligible studies and (2) screen full-text manuscripts to confirm eligibility. The screening form will be piloted on batches of 30 title/abstract pairings and refined until an inter-rater agreement, as measured by the Kappa statistic, of 0.80 or above is achieved [19]. Screening conflicts will be resolved through discussion between screeners or resolved by a third party (DD) if necessary during both stages of screening. Reasons for full-text study exclusion will be tracked and listed. Screeners will not be blinded to study authors, affiliated institutions, or journal titles.

**Table 2:** Article screening form

Study element	Meets criteria?		Reason for exclusion
Population: Does the study enroll a population of human adults with stroke?	Yes Unclear	No	1. Not an adult population. 2. Not a stroke population.
Intervention: Does the study involve stroke patients interacting with a mobile tablet device in response to a post-stroke deficit or complication?	Yes Unclear	No	3. Not a tablet-based therapy. 4. Survivors are not the primary tablet users.
Study Design: Does the manuscript report the results of a case study/series, cohort study, randomized or non-randomized controlled trial? If a study protocol or conference abstract, does it report the results of a study whose data is not otherwise available in a study manuscript?	Yes Unclear	No	5. Manuscript is a protocol/conference abstract with data available from a study manuscript. 6. Manuscript is a protocol/conference abstract with no reported data.

#### Data collection process

Two authors (MP and DJ) will independently extract key data items needed to describe the included studies and to answer the research questions stated above. A data extraction form (Additional file 2) will be used to guide data collection. Authors will compare and consolidate extracted information regularly to create a final data extraction form for each study. As the extraction process progresses, the data extraction form will be refined as necessary and any new pieces of information not collected in studies screened before changes occurred will be obtained by one of the study authors (MP or DJ). Study outcomes will be classified into one of three categories: barriers and adverse events, methodological challenges, and patient-reported outcomes. An assistive document (Table 3) will be used to help with the categorization of outcomes. Data extraction conflicts will be resolved through discussion between screeners or resolved by a third party (DD) if necessary. No effort will be made to collect missing information from study authors due to time constraints.

**Table 3:** Anticipated outcome categories of included studies

Outcome categorization	
Outcome category	Outcome sub-categories
Barriers and adverse events	Patient barriers, device barriers, environment barriers, solutions to barriers (proposed/attempted, and success if attempted), adverse events, other barriers or possible adverse events
Methodological challenges	Recruitment rate, adherence rate, retention rate (loss to follow-up), reasons for dropout, reasons for non-adherence, protocol deviations and/or revisions
Patient-reported outcomes	Ratings of perceived usefulness of intervention, ratings of intervention likability, other patient opinions.

### Data items

A wide variety of data items are needed to adequately answer the proposed research questions in context. Although every effort has been made to anticipate the broad extent of variables which will be collected, refinements to collected information will be made as the data collection process progresses if deemed necessary. Data items of interest fall into six categories: general study information, participant characteristics, intervention details, comparator details, outcomes, and setting and context. A full list of variables and clarifications where necessary can be found in Additional file 2.

### Outcomes and prioritization

There is no need to prioritize outcomes to accomplish the objective of the review. All data items and outcomes of interest will be collected and discussed and holistically answer research questions and meet the study objective.

### Risk of bias in individual studies

As per current scoping review guidelines, no formal risk of bias assessment will be performed for the included studies [14]. However, the potential impact of study design on individual study results will be discussed as the collected data items allow for an informed commentary.

### Data

#### Development of outcome themes

The categories and sub-categories listed above (Table 3) will serve to guide the thematic development of qualitative outcome information relevant to the three research questions. Relevant qualitative information (patient/caregiver/physician interviews, reaction, options, etc.) will be entered into spreadsheet software by one reviewer (MP) and grouped into an appropriate outcome category and sub-category (Table 3) after final consolidated data extraction sheets for each study have been approved by both extractors (MP and DJ). The frequency of encountered themes will be noted in the presentation of the final results.

#### Presentation of the final results

**Fig. 1:**
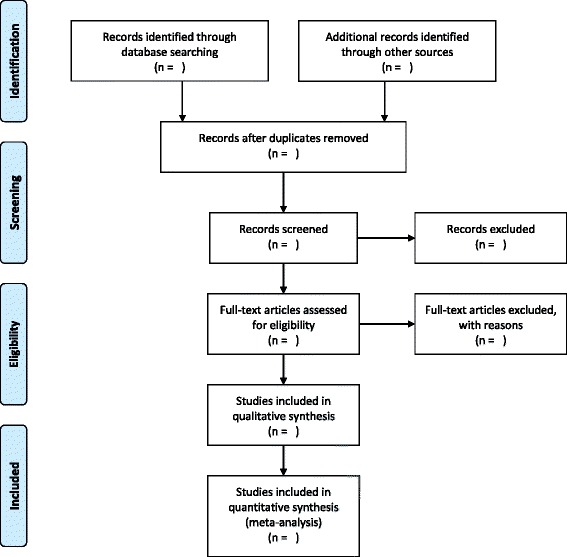
PRISMA flow diagram

Determining how to best present the results of a scoping review is an iterative process where the most logical approach becomes clearer as the data collection process comes closer to completion. Therefore, the following results outline is expected to be refined throughout the data extraction process. Search results will be summarized narratively and using the PRISMA flow diagram (Fig. 1) [20], and the characteristics of the final included studies will be summarized. The characteristics of the attempted intervention and administrative methods (therapy target, whether or not the therapy was performed with assistance, therapy setting, and whether the therapy was personalized) will be presented first to answer the first research question. This will be followed by lists of encountered barriers to care, adverse events, and other patient-reported outcomes organized by categories listed above (Table 3) to answer the second research question. A list of methodological challenges reported by the included studies will follow answering the third and final research question.

#### Meta-bias(es) and confidence in the cumulative evidence

There is no planned formal assessment of meta-biases or the confidence in the accumulated body of evidence. Instead, the impact of bias and strength of the evidence will be discussed based on the collected data points. More specifically, the strength of evidence supporting the feasibility of MTBTs following stroke will be covered in the discussion section of the final manuscript.

## Discussion

Answers to review questions will be proposed based on the accumulated evidence. Solutions to common challenges faced by patients and researchers will be proposed, and if appropriate, recommendations will be made to evaluate these solutions. Limitations of the reviewed studies will be discussed, and recommendations for improving the design of observational studies of MTBTs will be made. Gaps in research in terms of under-studied patient populations, interventional areas, and settings will be addressed. Recommendations will be made for small- and large-scale randomized controlled trials of MTBTs, and comments will be made on the appropriateness of conducting a systematic review and meta-analysis of completed MTBT trials.

## Additional files

Additional file 1: PRISMA-P checklist. (DOCX 28 kb)

Additional file 2: Data extraction form. (DOCX 13 kb)

## Abbreviations

App(s): Application(s)
MTBT: Mobile tablet-based therapy
RCT: Randomized controlled trial
