# Supplementary material for: Mobile tablet-based therapies following stroke: a systematic scoping review protocol of attempted interventions and the challenges encountered
Source: Syst Rev. 2017 Nov 2;6:219. doi: 10.1186/s13643-017-0620-6 (PMC5669017; doi:10.1186/s13643-017-0620-6)
Supplement: Supplementary file 2 — Data extraction form. (DOCX 13 kb) [file 13643_2017_620_MOESM2_ESM.docx]

**Data Extraction Form**

| **General Study Information** | |
| --- | --- |
| **Study Citation** |  |
| **Purpose/objective** |  |
| **Population** |  |
| **Study Design** |  |
| **Outcome ascertainment (self-report, interview, etc.)** |  |

| **Participants** | |
| --- | --- |
| **Inclusion/exclusion criteria** |  |
| **Sample Size** |  |
| **Stroke stage (Acute, chronic, mixed, or unknown)** | **Use time from stroke if it’s available, otherwise note stage.** |
| **Stroke type (Ischemic, hemorrhagic, mixed, unknown).** |  |
| **Average/Median age (SD/range/IQR)** |  |
| **Number/% female/male** |  |
| **Stroke severity (Include NIHSS or other measures if given)** |  |
| **Average/Median Time Post-Stroke (SD/range/IQR)** | **Time from stroke onset to initiation of MTBT (or if not explicitly stated, report time to MTBT study enrolment and make note of this).** |
| **Education Level** |  |
| **Number/% Familiar with touch-screen devices** |  |
| **Computer skill** |  |

| **Intervention** | |
| --- | --- |
| **Device make and model.** |  |
| **Therapy target (speech, fine-motor, etc.).** |  |
| **Therapy completed independently? If not, with whose assistance/oversight?** |  |
| **Used preexisting applications/software? It not, what was used and where did they originate from?** |  |
| **Dose/scheduling** |  |
| **Other important details regarding therapy approach and format**. |  |

| **Comparator(s)** | |
| --- | --- |
| **Description** |  |
| **Dose/schedule** |  |
| **Outcomes** | |
| **Barriers and Adverse events** |  |
| **Methodological Challenges** |  |
| **Patient Reported Outcomes** |  |
| **Setting and Context** | |
| **Therapy setting** |  |
| **Recruitment setting (if different from therapy setting)** |  |
| **Geographical location** |  |
| **Language** |  |
